# A bio on bioelectrochemical cells: a conversation with Nicolas Mano

**DOI:** 10.1038/s41467-020-19776-9

**Published:** 2020-12-14

## Abstract

Dr Nicolas Mano is a Senior Researcher at Centre de Recherche Paul Pascal, France. His research interests include (bio)electrochemistry, biosensors, biofuel cells, enzymes engineering, and the use of carbonaceous materials for electrodes. His aim is to develop approaches where biochemical fuels can be converted into electricity and applied into bioelectrochemical applications. In this conversation, he is discussing the advancements in the field of biofuel cells in the past ten years and look ahead at future developments.

**Figure:**
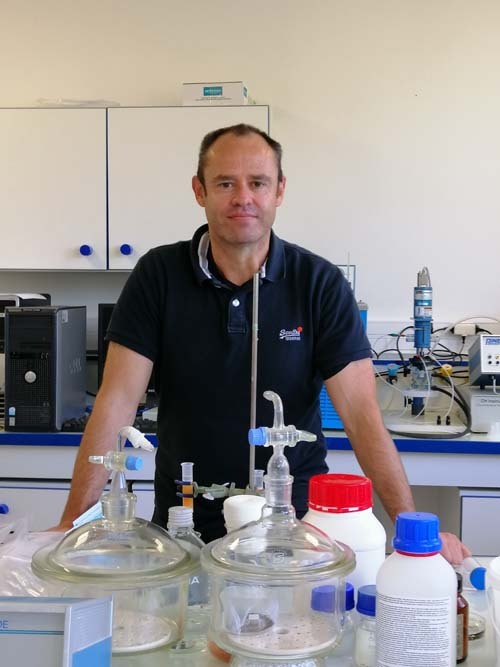
Dr Nicolas Mano

How did you get into the field of electrocatalysis/biofuel cells? Why did you want to pursue this research area?

I was trained as an environmental chemist and discovered electrochemistry and electrocatalysis during my master’s and Ph.D. under the supervision of Prof Alexander Kuhn at the University of Bordeaux. At that time, I had my first glimpse at bioelectrochemistry while working with enzymes. It was exciting to work with enzymes, and I realized the many possibilities that opened up using them. At that point, I knew it would be my major field of research. Afterward, I moved to the University of Texas at Austin and had the chance to work with Prof Adam Heller. There I learned much more about bioelectrochemistry, glucose biosensors, and the challenges associated with the connection of redox centers of enzymes to electrode surfaces to design efficient biosensors. While I was at UT Austin, I discovered the work of glucose/O_2_ biofuel cell which was about to be stopped due to the lack of stable cathodic enzymes in the presence of chloride and neutral pH. And while Prof Heller hired me to work on DNA sensors, this topic captivated me and I pursued the work on biofuel cells using a different cathodic enzyme, which was just reported by Prof Ikeda at the University of Tokyo. Back in France, I first launched research projects on molecular biology to produce enzymes and be able to control their purities/activities and on materials chemistry to elaborate/ optimize electrodes materials and surfaces. Since then, I have been working on various subthemes of electrochemistry with a special emphasis on bioelectrochemistry. This is a fascinating and challenging field of research requiring interdisciplinary knowledge ranging from molecular biology, modelling to material science.

Could you briefly summarize the work in this paper “Engineering hybrid nanotube wires for high-power biofuel cells”

In this work, we described a strategy to assemble and align carbon nanotubes (CNTs) into porous microwire electrodes. We tested these new microwires for the enzymatic reduction of oxygen. Compared to conventional carbon fibers, the new design exhibited tenfold higher performances. Used in glucose/oxygen biofuel cells (BFCs), it permits increasing the power densities four times compared to BFCs made with carbon fibers. Such characteristics result from the unique features provided by these microwires. The porosity allows increasing the specific surface, maximizing the number of immobilized enzymes and therefore the current density. The cylindrical geometry maintains the geometric surface as small as possible. Both features allow overcoming mass transport limitations encountered in physiological media, where the diffusion coefficient and concentration of substrates are low. The size and the electrical properties of carbon nanotubes enhance the electron transfer while favoring better wiring of enzymes. Finally, the orientation of the CNTs reinforces the structural stability of the entire system.

What motivated you to submit your work in newly launched Nature communications?

The editorial team of another Nature journal offered us the opportunity to transfer our manuscript to a new journal. At that time, we knew nothing about Nature communications. But we had no hesitation. Nature journal have excellent reputations and there was no doubt in our minds this would be a selective journal. In addition, we were not limited by the number of pages allowing us to explain in detail the rationale behind our work.

In a collaborative project, how do you decide where to publish?

For interdisciplinary work, for example gathering biologists, physicists, and materials scientists, I feel there is always a compromise between the journal core expertise/reputation and the primary audience to be reached. But a paper well written will ever come to its intended audience and excite the curiosity of others.

How do you feel the field of electrocatalysis and fuel cells has changed in the past decade since you published your article with us?

In the last decade, we observed the emergence of electrochemical systems of higher complexity and sophistication. For example, electrodes are becoming more sophisticated bearing hierarchical porosity and being more flexible, bendable, and stretchable. We also noted the appearance of self-healing electrodes. With the aim to provide for a sustainable future, we have seen a dramatic increase in papers on biophotoelectrochemistry for solar energy harvesting using photosynthetic proteins, H_2_ production from solar-based systems, or CO_2_ reduction in green solvents, for example. Because the efficiency of those systems depends on many interrelated parameters, we also witnessed the use of combined analytical techniques.

From your point of view, where do you see the field of electrocatalysis in the next 5 years?

Our world is addicted to fossil fuels. “I feel that electrocatalysis is still underrated and I would not be surprised if its applications spread in the next few years to shift from an “oil-based world” to a “bioresource-based world”. I can see (bio)-electrochemistry/electrocatalysis playing a key role in manufacturing useful and environmentally benign molecules, sustainable materials, or in clean energy. Electrocatalysis should also drive progress in the emerging field of sustainable catalysis where bioelectroenzymatic synthesis in aqueous media bears a great promise. All this without compromising the quality of life in the year to come. Besides, the use of electrocatalysis will also be accelerated by developing new multidisciplinary approaches.

What advice would you give to early career researchers looking to publish papers in a selective journal?

First, I would say we are too often evaluated on the number of papers we published in selective journals. One can make excellent science and have a tremendous impact on a community without publishing in selective journals.

Then, to publish in a selective journal, I feel the work should address an important scientific topic of interest for various scientific communities and show clear scientific advances of the field. Data should be carefully analysed and compared/discussed with the existing literature. But good science also need to be effectively communicated.  Therefore, the writing has tremendous importance to convey a good message and present data clearly and concisely. This is challenging as it requires experience and skills. I would suggest reading tutorials, getting help from senior scientists, and follow suggestions provided by the journal’s editors.

*The interview was conducted by Team Manager & Senior Editor Dr. Prateek Dongare.*

